# Supplementary material for: Efficient Suppression of Abdominal Aortic Aneurysm Expansion in Rats through Systemic Administration of Statin-Loaded Nanomedicine
Source: Int J Mol Sci. 2020 Nov 18;21(22):8702. doi: 10.3390/ijms21228702 (PMC7699030; doi:10.3390/ijms21228702)
Supplement: Supplementary file 1 [file ijms-21-08702-s001.pdf]

Supplementary Materials

Efficient Suppression of Abdominal Aortic Aneurysm Expansion in Rats by Systemic Administration of Statin-Loaded Nanomedicine

Natsumi Fukuhara [1], Yuto Honda [2,3], Nao Ukita [3], Makoto Matsui [2], Yutaka Miura [2,3,*] and Katsuyuki Hoshina [1,*]

[1] Division of Vascular Surgery, Department of Surgery, Graduate School of Medicine, The University of Tokyo, 7-3-1 Hongo, Bunkyo-ku, Tokyo 113-8655, Japan ; FUKUHARAN-SUR@h.u-tokyo.ac.jp (N.F.), traruba@gmail.com (K.H.)

[2] Laboratory for Chemistry and Life Science, Institute of Innovative Research, Tokyo Institute of Technology, 4259 Nagatsuta-cho, Midori-ku, Yokohama, Kanagawa, 226-8503, Japan ; honda.y.aj@m.titech.ac.jp (Y.H.), matsui.m.ad@m.titech.ac.jp (M.M), miura.y.ai@m.titech.ac.jp (Y.M.)

[3] Department of Life Science and Technology, School of Life Science and Technology, Tokyo Institute of Technology, 4259 Nagatsuta-cho, Midori-ku, Yokohama, Kanagawa 226-8503, Japan; ukita.n.aa@m.titech.ac.jp (N.U.)

* Correspondence: miura.y.ai@m.titech.ac.jp (Y.M.), traruba@gmail.com (K.H) ; Tel.: +81-45-924-5225 (Y.M.), +81-35-800-8653 (K.H)

Supplementary Figures

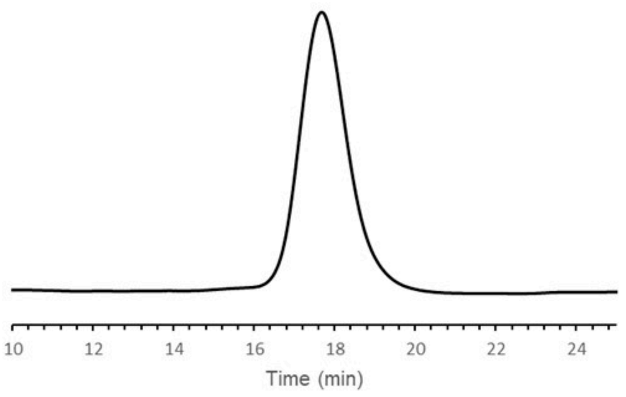

Figure S1. GPC chart of PEG-P[Lys]$_{45}$. Column: SuperdexTM 200 Increase 10/300 GL, eluent: 10mM phosphate buffer (pH7.4) containing 500 mM NaCl, flow rate: 0.5 mL/min, temperature: room temperature, detection: absorbance at 220 nm.

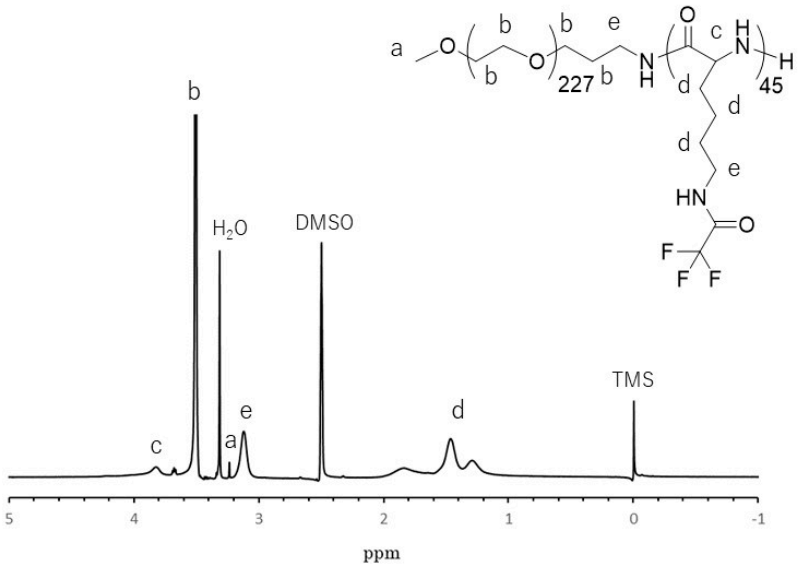

Figure S2. ^{1}H-NMR spectrum of PEG-P[Lys(TFA)]$_{45}$ (solvent: DMSO).

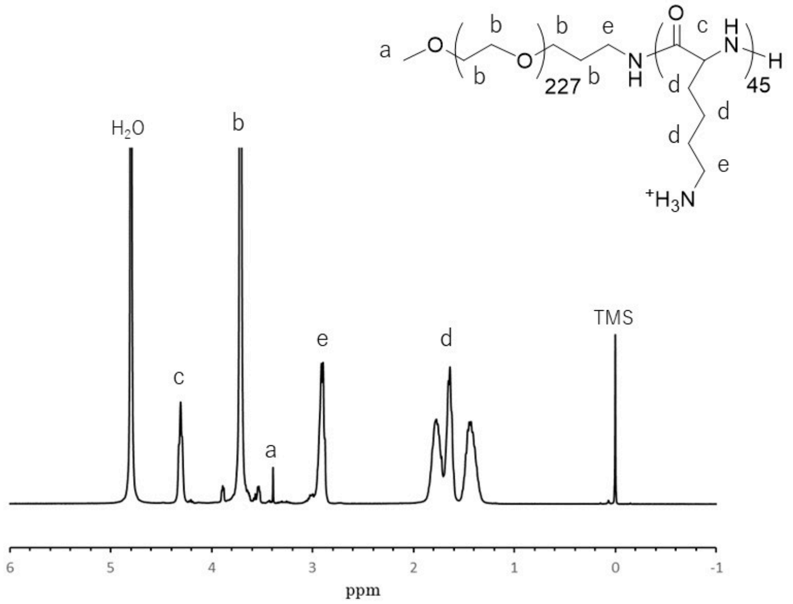

Figure S3. ^{1}H-NMR spectrum of PEG-P[Lys(TFA)]$_{45}$ (solvent: DMSO).

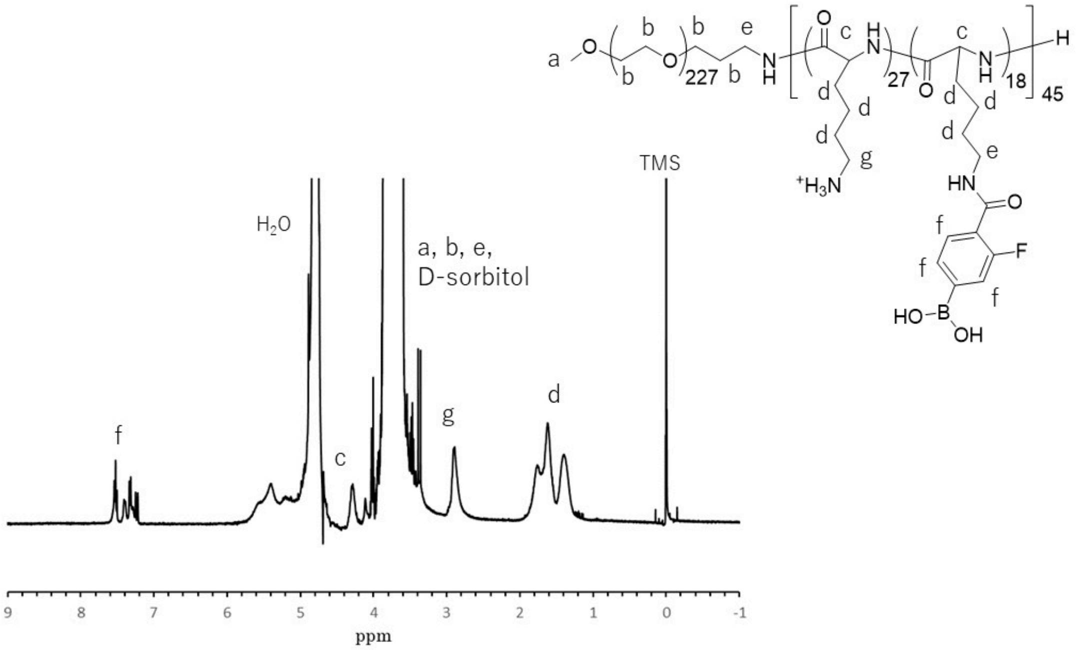

Figure S4. ^{1}H-NMR spectrum of PEG-P[Lys(FPBA)]$_{45}$ (solvent: D_2O with 90 mg/mL of D-sorbitol)).

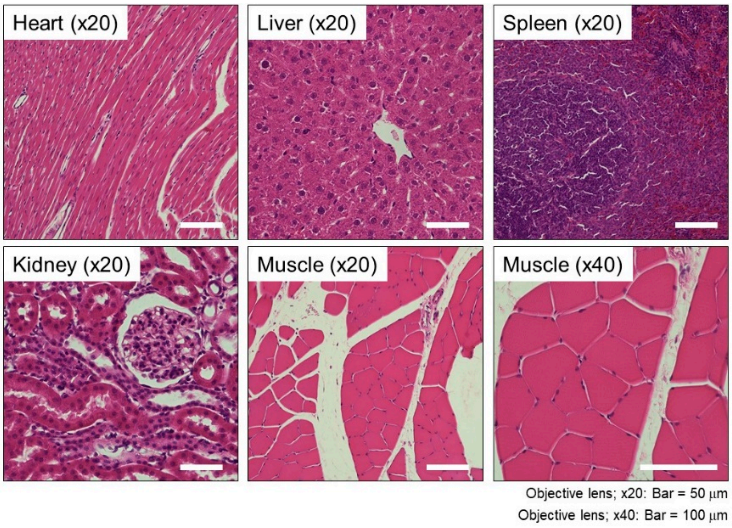

Figure S5. Photomicrographs of organs after PS/m treatment. Hematoxylin and eosin-stained heart, liver, spleen, kidney, and muscles. Scale bars represent 50 µm in x20 images and 100 µm in x40 image.
